# Premature cognitive decline in a mouse model of tuberous sclerosis

**DOI:** 10.1111/acel.14318

**Published:** 2024-08-27

**Authors:** J. Krummeich, L. Nardi, C. Caliendo, D. Aschauer, V. Engelhardt, A. Arlt, J. Maier, F. Bicker, M. D. Kwiatkowski, K. Rolski, K. Vincze, R. Schneider, S. Rumpel, S. Gerber, M. J. Schmeisser, S. Schweiger

**Affiliations:** ^1^ Institute of Human Genetics University Medical Center of the Johannes Gutenberg University Mainz Mainz Germany; ^2^ Institute of Anatomy University Medical Center of the Johannes Gutenberg University Mainz Mainz Germany; ^3^ Institute of Physiology University Medical Center of the Johannes Gutenberg University Mainz Mainz Germany; ^4^ Department of Biochemistry University of Innsbruck Innsbruck Austria; ^5^ Leibniz Institute of Resilience Research Mainz Germany; ^6^ Institute of Molecular Biology Mainz Germany; ^7^ Present address: Bioscientia Institut für Medizinische Diagnostik GmbH Humangenetik Ingelheim Germany; ^8^ Present address: Institute for Genomic Statistics and Bioinformatics University of Bonn Bonn Germany

**Keywords:** aging, IGF2, mTOR, neurodegeneration, premature cognitive decline, rescue

## Abstract

Little is known about the influence of (impaired) neurodevelopment on cognitive aging. We here used a mouse model for tuberous sclerosis (TS) carrying a heterozygous deletion of the *Tsc2* gene. Loss of *Tsc2* function leads to mTOR hyperactivity in mice and patients. In a longitudinal behavioral analysis, we found premature decline of hippocampus‐based cognitive functions together with a significant reduction of immediate early gene (IEG) expression. While we did not detect any morphological changes of hippocampal projections and synaptic contacts, molecular markers of neurodegeneration were increased and the mTOR signaling cascade was downregulated in hippocampal synaptosomes. Injection of IGF2, a molecule that induces mTOR signaling, could fully rescue cognitive impairment and IEG expression in aging *Tsc2* ^*+/−*^ animals. This data suggests that TS is an exhausting disease that causes erosion of the mTOR pathway over time and IGF2 is a promising avenue for treating age‐related degeneration in mTORopathies.

## Graphical abstract

Tuberous sclerosis (TS) is a progressive neurodevelopmental disorder caused by mutations in *Tsc1* or *Tsc2* genes, leading to hyperactivity of the mTOR pathway. Using a conventional *Tsc2^+/−^* model, we observed a premature decline of cognitive functions and a reduction of immediate early gene expression, that could be rescued by IGF2. Our data suggest that TS is an exhausting disease that causes erosion of the mTOR pathway over time and IGF2 is a promising avenue for treating age‐related degeneration in mTORopathies.

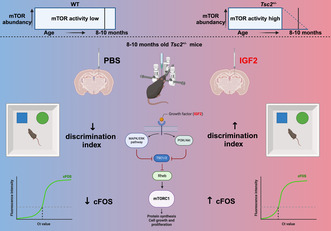

**Abbreviations**
ASD: autism spectrum disorder
CA1: cornu ammonis 1
CA3: cornu ammonis 3
cDNA: complementary DNA
CTB: Cholera Toxin Subunit B
Ctx: cortex
DG: dentate gyrus
DI: discrimination Index
EMB: episodic memory battery
GO: Gene Ontology
Hip: hippocampus
ID: intellectual disability
IEG: immediate early gene
IGF2: Insulin‐like growth factor 2
mRNA: messenger ribonucleic acid
mTOR: mammalian target of Rapamycin
NORT: novel object recognition test
NSC: neural stem cell
PBS: phosphate buffered saline
PFC: prefrontal Cortex
qRT‐PCR: real time quantitative PCR
TAND: Tsc‐associated neuropsychiatric disorder
TS: Tuberous Sclerosis
Tsc1: TSC Complex Subunit 1
Tsc2: TSC Complex Subunit 2
WT: wildtype

## 1 INTRODUCTION

Tuberous sclerosis (TS) is a multi‐system, neurodevelopmental disorder caused by heterozygous mutations in either *Tsc1* or *Tsc2* genes, two negative regulators of mammalian target of rapamycin (mTOR) activity. mTOR is a key regulator of local protein synthesis in dendrites and synapses. Clinical symptoms of TS are thought to be caused by mTOR hyperactivity (summarized in Salussolia et al., 2019). Symptoms include multiple skin aberrations, predisposition to cancer, and Tsc‐associated neuropsychiatric disorder (TAND). While skin and some neoplastic lesions (e.g., cortical tubers) occur in the fetal to neonatal period, epilepsy develops in infancy (Salussolia et al., 2019). The TAND spectrum is very diverse and, among others, includes autism spectrum disorder (ASD) and intellectual disability (ID). It comprises a significant source of morbidity (summarized in Mizuguchi et al., 2021).

Clinically, TS is highly variable, ranging from very severely affected to patients with hardly recognizable signs of disease in around 10% of the cases with molecularly confirmed TS (Uysal & Sahin, 2020). Appearance of TAND symptoms follows a longitudinal trajectory with epilepsy occurring in 80% of the TS cases and often being the first neurological symptom recognized mostly in the first year of life (Salussolia et al., 2019). ASD‐like symptoms occur as late as at 7–8 years of age together with ID, while mood disorders are usually seen in adult patients (de Vries et al., 2018). Occurrence of epilepsy has a substantial influence on the prognosis and the development of other TAND symptoms (Capal et al., 2017). However, neurocognitive symptoms also develop independently of epilepsy in patients and animals (Crino et al., 2006; Ehninger et al., 2008).

While both, *Tsc1* and *Tsc2*, are negative regulators of mTOR activity and, when mutated, lead to mTOR hyperactivity in neural tissue, *Tsc2* mutations generally cause more severe phenotypes (Curatolo et al., 2015; Dabora et al., 2001; Henske et al., 2016) and also a larger spectrum of TAND symptoms in patients (de Vries et al., 2018; Magri et al., 2013). Rather than disease development as a static process, the stepwise establishment of TAND symptoms suggests a dynamic change of brain network stages slowly moving into pathology with vulnerable windows at different phases of neurodevelopment. Understanding the underlying molecular mechanisms of these dynamics is an important tool for the development of successful interventions.

ID is one of the most important prognostic factors in TS and, when present, is associated with a high burden for patients and carers. To study the disease processes that lead to cognitive impairment, we have used a conventional mouse model carrying a heterozygous deletion in the *Tsc2* gene in a longitudinal behavioral analysis focusing on cognitive performance. Because, other than in models carrying mutations in *Tsc1*, in models with *Tsc2* aberrations, epilepsy is not found (von der Brelie et al., 2006) and not even predisposition to hyperexcitability is seen at early postnatal stages (Bassetti et al., 2020), this model allowed studying the occurrence of cognitive impairment independent of epileptic potential.

We are here using a conventional *Tsc2* ^*+/−*^ model in a longitudinal setting to study onset of cognitive impairment and link it to underlying molecular changes. While first signs of ASD were found in the *Tsc2* ^*+/−*^ animals at early adulthood (3 months of age), cognitive performance remained unaffected until late stages. Only at 8–10 months of age, subtle cognitive aberrations were observed, suggesting a premature cognitive decline in the *Tsc2* ^*+/−*^ animals rather than a congenital defect in intellectual capability. Surprisingly, proteomic analysis showed a significant reduction of large parts of the mTOR signaling cascade at this age. Causality of this for the observed symptoms was demonstrated by a full rescue through stereotypic hippocampal injection of IGF2. Our data points towards a consumptive loss of molecules of the mTOR signaling cascade with time in animals with hyperactive mTOR over lifetime leading to premature cognitive decline.

## 2 MATERIALS AND METHODS

### 2.1 Animals

Heterozygous *Tsc2* mice were purchased from the Jackson Laboratory (B6;129S4‐*Tsc2* ^*tmDjk*^/J; JAX #004686) containing a neo‐cassette targeted disruption of the second coding exon in the *Tsc2* gene. PCR genotyping was performed by simultaneous amplification of both wildtype *Tsc2* and mutant alleles using three primers (H162‐CAAACCCACCTCCTCAAGCTTC, H163‐AATGCGGCCTCAACAATCG, and H164‐AGACTGCCTTGGGAAAAGCG). Products (H163/H162 86 bp for the wildtype allele and H164/H162 105 bp for the mutant allele) were analyzed on a 3% agarose gel. For the immunohistochemistry of synaptic proteins and the dendritic spine density analyses, *Tsc2* heterozygous mice were crossed to mice expressing GFP in neurons (Feng et al., 2000). Hemizygous Thy1‐EGFP mice were purchased from the Jackson Laboratory (JAX #007788). The mice were kept under specific‐pathogen‐free conditions in groups of two to four mice per cage on a 12‐h light/12‐h darkness cycle with water and food provided ad libitum.

### 2.2 Behavioral assays

All experimental procedures were performed in accordance with institutional animal welfare guidelines and were approved by the state government of Rhineland‐Palatinate, Germany (G15‐1‐068, G20‐1‐117, G20‐1‐144). For behavioral analyses, 3–4 months and 8–10 months old male *Tsc2* ^*+/−*^ mice and age‐matched wildtype littermates were used. When obtaining behavioral profiles, the animals were tested once for each test. All tests were performed between 08:00 am and 2:00 pm. Behavioral data are presented as the mean ± standard error of the mean (SEM). Prism 8 software (San Diego, CA, USA) was used for statistical analyses, and *p* < 0.05 was considered statistically significant. Outliers were identified using the ROUT function (*Q* = 1%) in GraphPad Prism. Normality of groups was checked by using D'Agostino‐Pearson tests. All two‐group comparisons were calculated using either *t* test when data followed a Gaussian distribution or the Mann–Whitney test when data were not normally distributed.

### 2.3 Novel object recognition test

For the first training phase of the Novel object recognition test (NORT), mice were placed into an arena (40 cm wide × 40 cm deep × 40 cm high) and presented with two identical objects. Mice were allowed to explore the arena and the objects for 5 min before being removed for a 24‐h interval spent in their home cage. On day 2, in a second 5‐min training phase, the same two identical objects were presented, followed by another 24‐h retention interval. In a 5 min test phase on day 3 (24‐h NORT) or day 9 (7 days NORT), one of the objects was replaced by a novel object, and the time mice spent exploring the objects was recorded by camera and analyzed manually by using BORIS v. 6.3.9 software. The discrimination index (DI) was calculated as: (time novel object) − (time familiar object)/(time novel object + time familiar object).

### 2.4 Episodic memory battery

The episodic memory battery consists of four tests (novel‐object‐recognition, object‐place‐recognition, object‐context recognition, object‐place‐context recognition), all structured similarly to the typical novel object recognition test. Each test was performed on four consecutive days with new pairs of objects every day. The individual tests differ in that familiar or novel objects are presented not only at a specific time but also at a specific location within a particular context. The time between each training and testing phase was 5 min. The time mice spent exploring the objects was recorded by a camera and analyzed manually using BORIS v. 6.3.9 software. The DI was calculated as: (time novel object) – (time familiar object)/(time novel object + time familiar object).

### 2.5 Neuroanatomical tract‐tracing

To retrogradely label neurons within the perforant pathway, 200 nL of 5 μg/μl fluorescently conjugated Cholera Toxin Subunit B (CTB; Cholera Toxin Subunit B Alexa Fluor™ 488 Conjugate #C34775, Alexa Fluor™ 555 Conjugate #C34776, Alexa Fluor™ 647 Conjugate #C34778) (Invitrogen, Waltham, MA) were injected unilaterally into the dorsal hippocampus (Alexa Fluor 488: −1.9 mm Anterior–Posterior, 1.3 mm Medio‐Lateral, −1.8 mm Dorso‐Ventral to bregma), ventral hippocampus (Alexa Fluor 555: −3.5 mm Anterior–Posterior, 3.0 mm Medio‐Lateral, −3.3 mm Dorso‐Ventral to bregma) and the entorhinal cortex (Alexa Fluor 647: −4.2 mm Anterior–Posterior, 3.8 mm Medio‐Lateral, −5.0 mm Dorso‐Ventral to bregma) according to the Paxinos and Franklin's mouse brain atlas. 8–10 months old *Tsc2* ^*+/−*^ male mice were anesthetized with isoflurane (2.5% in medical oxygen) and placed in a stereotaxic frame. A glass micropipette was used for infusion, the infusion rate was 20 nL/min. The glass micropipette was left in place for 5 min to limit the nonspecific spread of the tracer. After wound closure, animals were allowed to recover for 7 days before tissue harvest.

Brains were dissected from skulls and post‐fixed with 4% PFA at 4°C overnight and then immersed in PBS at 4°C before sectioning. Using a vibratome (Leica VT1200, Leica Microsystems, Nussloch, Germany), 70 μm coronal brain sections were cut from posterior to anterior and stored in PBS with 0.01% sodium azide at 4°C. After counterstaining with DAPI (4′,6‐Diamidino‐2‐phenylindol; 1 μg/mL), sections were mounted on slides with Fluoromount (Sigma‐Aldrich, St Louis, MO). Each section was imaged at 20× air objective (HCX PL APO CS 20.0 × 0.70 DRY UV) using a Leica SP5 confocal laser scanning microscope (Leica TCS SP5, Leica Camera AG, Wetzlar, Germany) with parameters adjusted based on the intensity of expression and background fluorescence. Labeled CTB+ neurons in each layer were identified automatically using Imaris software (Oxford instruments) and analyzed using Matlab (MathWorks).

### 2.6 Immunohistochemistry of synaptic proteins

Nine months old Thy1‐GFP^+^/*Tsc2* wildtype (*n* = 6) and Thy1‐GFP^+^/*Tsc2* ^*+/−*^ (*n* = 6) male mice were transcardially perfused with 1× PBS, followed by 4% PFA in PBS. Brains were post‐fixed overnight in PFA and further cut at the vibratome (Leica VT 1000 S, Leica Microsystems, Nussloch, Germany). 40 μm thick slices obtained at the bregma point −1.91 mm Anterior–Posterior were used. The brain slices were washed three times for 2 min in 1× PBS. Afterwards, they were incubated for 1 h at room temperature in blocking solution (20% BSA, 0.1% Triton‐X in 1× PBS). Finally, they were incubated overnight at 4°C with primary antibodies in 10% BSA, 0.1% Triton‐X in 1× PBS. The primary antibodies and concentrations reported below were used in this study: DAPI (62248, ThermoScientific, 1:1000), anti‐Vglut1 (guinea pig, 135304, Synaptic Systems, 1:250), anti‐Homer1 (rabbit, 160023, Synaptic Systems, 1:250), anti‐Vgat (guinea pig, 131004, Synaptic Systems, 1:250) and anti‐Gephyrin (rabbit, 147018, Synaptic Systems, 1:250). Slices were washed again three times in 1× PBS for 2 min and later incubated with the corresponding secondary antibodies for 75 min at room temperature in 10% BSA, 0.1% Triton‐X in 1× PBS. The secondary antibodies and concentrations reported below were used in this study: Alexa568‐conjugated anti‐guinea pig (goat, A‐11075, Thermo‐Fisher, 1:500), Alexa647‐conjugated anti‐rabbit (donkey, A‐31573, Thermo‐Fisher, 1:1000). DAPI was finally added to the solution containing the secondary antibody for 15 min at room temperature. Another washing step followed (Three times for 5 min in 1× PBS) before mounting the slices with Fluoromont‐G (0100‐01, SouthernBiotech). Images were acquired with a confocal microscope (Leica TCS, SP8, 10× and 63× objectives). The images acquired with the 63× objective were further analyzed with the synapse counter plugin of Fiji. Raw data were normalized as percentage of control wildtype values. The analysis of the data obtained was then performed with GraphPad Prism 8 (GraphPad Software, Inc.). Normal distribution of the data sets was ascertained through the Shapiro–Wilk test. Student's *t* test was performed, taking *p* < 0.05 threshold for statistical significance. Results are shown as percentage of control wildtype values as mean ± SEM.

### 2.7 Dendritic spine detection and analysis

From the same brains used for the immunohistochemistry of synaptic proteins, 250 μm thick brain slices were obtained starting at the bregma point −1.91 mm anterior–posterior. The brain slices were washed three times for 2 min in PBS 1× and mounted with Fluoromont‐G (0100‐01, SouthernBiotech). Due to the strong signal deriving from the expression of GFP, images could be directly acquired through a confocal microscope (Leica TCS SP8, Leica Camera AG, Wetzlar, Germany) with a 63× objective, 488 laser. Secondary, apical dendrites in the stratum radiatum of the CA1 area of the hippocampus were acquired with a *Z* stack of 0.2 μm. The pictures went through deconvolution with the Lightning software and were then converted to IMARIS data files (version 9.7, Bit‐ plane Inc., St. Paul, MN). Dendritic shafts and spines were reconstructed manually using the Filament module of the IMARIS software. Spine density was calculated as sum of the total number of spines divided the dendritic length. For each animal 10–15 dendritic spines were analyzed. The values were averaged to express the spine density/10 μm for each animal. The Shapiro–Wilk test was performed to assess normal distribution of the data. Student's *t* test was performed, taking *p* < 0.05 as threshold for statistical significance. Final results are shown as percentage of control wildtype values as mean ± SEM.

### 2.8 BrdU injection and immunohistochemistry

For quantitative analysis of adult neurogenesis in vitro, 10 months old male *Tsc2* ^+/−^ mice and their corresponding wildtype littermates got injected with 1 mg BrdU/kg bodyweight over a period of 3 days intraperitoneally. One week prior to the Bromdesoxyuridin (BrdU) injection, we enriched the environment of the analyzed group with a running wheel. Three weeks post‐injection, mice were sacrificed by perfusion using 4% paraformaldehyde (PFA) and the tissue was stored at 4°C till further use. Following transcranial perfusion, brains were embedded in 4% low melt agarose and further cut serially in 40 μm coronal sections using the Leica VT 1000s vibratome (Leica Camera AG, Wetzlar, Germany). Following three PBS washing cycles, the brain sections were incubated for 30 min in 2 N HCL at 37°C, followed by a 10 min incubation in borate buffer. Prior to the antibody incubation, the brain sections were blocked using a 10% goat serum (NGS) in 1×PBS‐T (0.2%). Primary BrdU (Abcam, Cambridge, United Kingdom, ab6326), Neuronal Nuclei (NeuN) (Abcam, ab177487) and Ki67 (Abcam, ab16667) antibodies were diluted in 3% NGS in 1×PBS‐T. Sections were incubated with primary antibodies in blocking solution at 4°C overnight. Following three PBS washing cycles, the brain sections were incubated with species‐specific fluorophore‐conjugated secondary antibodies at room temperature for 1 h, the nuclear counterstain DAPI (4′,6‐diamidino‐2‐phenylindole) was used. Immunohistochemically stained brain sections were imaged using a Leica TCS SP8 confocal laser scanning microscope (Leica Camera AG, Wetzlar, Germany). The hippocampal dentate gyrus (DG) was imaged using an oil‐supported 63× objective. The fluorescence spectrum between 400 and 647 nm was used and detected by 2 PMTs and 1 HyD enabling the visualization of different emission spectra. BrdU^+^/NeuN^+^ as well as Ki67^+^ cells were quantified and statistically analyzed using an unpaired *t* test.

### 2.9 IGF2 administration

A single dose of recombinant mouse IGF2 protein (R&D Systems, #792‐MG) was stereotactically injected into the hippocampus of 8–10 months old *Tsc2* ^*+/−*^ mice and wildtype littermates after the training phase 2 (day 2) of the 7 days NORT (Figure 6a). Two hours after training, mice were treated with carprofen (5 mg/kg) for analgesia and anesthetized with 1.5%–2% isoflurane. To avoid hypothermia, mice were kept on an electric blanket during the entire duration of surgery. In addition, ointment (Bepanthen, Bayer, Leverkusen, Germany) was applied on the eyes to prevent their dehydration. While the animals were under deep anaesthesia, a 0.5 cm longitudinal incision was made with a scalpel above the position of the bregma. Mice were fixed in a stereotaxic frame (Kopf Instruments, Tujunga, CA, USA) and 1 μL of IGF2 (250 ng/μL in PBS containing 0.1% BSA) were administered into the dorsal hippocampus of both hemispheres (−1.9 mm Anterior–Posterior, 1.3 mm Medio‐Lateral, 1.8 mm Dorso‐Ventral to bregma). Control animals were injected with 1 μL of PBS+ 0.1% BSA. Seven days after surgery, mice performed the test phase of the 7 day NORT, and were subsequently sacrificed for brain removal.

### 2.10 RNA extraction and quantitative real‐time PCR (qPCR)

Total RNA from total hippocampus was isolated using Trizol reagent (Sigma‐Aldrich, St Louis, MO). The RNA was treated with DNase and reverse transcribed into cDNA using PrimeScript™ RT Master Mix. For the quantification of IEG *cfos* and *zif268* expression, quantitative real‐time PCR assays were performed in triplicate using SYBR Premix Ex Taq™, and specific primers to mouse *cfos* (forward: GGCTGCACTACTTACACGT; reverse: TGCCTTGCCTTCTCTGACTG), mouse *zif268* (forward: ATGAGAAGGCGATGGTGGAG; reverse: CTCACGAGGCCACTGACTAG) and *gapdh* (forward: CATCACTGCCACCCAGAAGACTG; reverse: ATGCCAGTGAGCTTCCCGTTCAG) was used as an internal control. Real‐time PCR was done using a StepOnePlus™ Real‐Time PCR Cycler.

### 2.11 Isolation of synaptosomes

Mouse brains were isolated after cervical dislocation and hippocampi were dissected on ice. To obtain hippocampal synaptosomal fractions, Syn‐PER™ synaptic protein extraction reagent (Thermo Scientific™) was used by following manufacturer's protocol. In brief, mouse brain hippocampi were homogenized in Syn‐PER™ reagent including Halt™ protease and phosphatase inhibitor cocktail (1:100 ratio; Thermo Scientific™) by using Dounce tissue homogenizer (KIMBLE®). For uniform processing, 3 strokes with pestle A and 10 strokes with pestle B were performed. The mixture was transferred to a 1.5 mL tube and centrifuged for 10 min at 1200*g* and 4°C. The supernatant was centrifuged at 15,000*g*, 4°C for 20 min. The resulting pellet contained the synaptosomal enriched fraction. This pellet was resuspended in an appropriate volume of pre‐cooled Syn‐Per™ reagent including Halt™ protease and phosphatase inhibitor cocktail and stored at −80°C until further analysis.

### 2.12 Proteome extraction and tryptic digestion

Proteins were extracted from the hippocampus of six wildtype mice and six heterozygous *Tsc2* ^*+/−*^ mice at the age of 8–10 months using a sodium deoxycholate (SDC) containing lysis buffer (van Pijkeren et al., 2023). The samples were lysed by addition of 300 μL SDC buffer (2% w/v SDC, 100 mM triethylammonium bicarbonate (TEAB), pH 8.3). The samples were heated for 5 min at 98°C and sonicated at room temperature (RT) (Branson Ultrasonics Sonifier Model 250 CE, Thermo Fisher Scientific, parameters: 1× 10 s, constant duty cycle, output control: 2). Total protein amounts were quantified using the micro bicinchoninic acid (BCA) protein assay (Micro BCA™ Protein Assay Kit, Thermo Scientific, Germany) following the vendors protocol. From each sample, a volume containing 100 μg of total protein was transferred to a new reaction tube and made up to a final volume of 100 μL with 100 mM TEAB (pH 8.3). For reduction, 1.05 μL of a dithiothreitol containing reduction buffer (1 M DTT, dissolved in 100 mM TEAB, pH 8.3) was added and samples were incubated for 30 min at 55°C and 800 rpm on a thermo‐shaker. For alkylation, 4.6 μL of iodoacetamide (IAA) containing alkylation buffer (0.5 M IAA, dissolved in 100 mM TEAB, pH 8.3) were added and samples were incubated for 30 min in the dark, followed by addition of 1.2 μL of reduction buffer to quench the alkylation reaction. Afterwards, 102.2 μL of 100 mM TEAB was added and proteins were digested for 16 h at 37°C using 5 μg of trypsin (dissolved in trypsin resuspension buffer, Promega, Walldorf, Germany). Tryptic digestion was stopped by addition of 2.5 μL 100% formic acid, and the samples were centrifuged for 5 min (16,000 g, RT) to remove the precipitated SDC. Supernatants were used for reversed phase solid phase extraction (RP‐SPE).

### 2.13 Reversed phase solid phase extraction (RP‐SPE)

Samples were purified by RP‐SPE prior to LC–MS analysis using OASIS HLB cartridges (Oasis HLB, 1 cc Vac Cartridge, 30 mg Sorbent, Waters, Manchester, UK) and a pressure manifold (Waters SPE Manifold, Waters, Manchester, UK). SPE cartridges were activated with 1 mL of 100% methanol (MeOH), followed by 1 mL of 95% ACN, 1% FA, and equilibrated with 1 mL of 1% FA. The samples were adjusted to a final volume of 1 mL and a final concentration of 1% FA, loaded on SPE cartridges and washed three times with 1 mL 1% FA. The peptides were eluted with 1 mL of 70% ACN, 1% FA. The eluates were evaporated to dryness using an Eppendorf Concentrator Plus (Eppendorf, Hamburg, Germany) and stored at −80°C.

### 2.14 Proteome analysis by LC–MS/MS

Dried peptide samples were dissolved in 80 μL of 0.1% FA, and 1 μL of the samples were injected into a nano‐ultra pressure liquid chromatography system (Dionex UltiMate 3000 RSLCnano pro flow, Thermo Scientific, Bremen, Germany) coupled via electrospray ionization (ESI) to a tribrid Orbitrap mass spectrometer (Orbitrap Fusion, Thermo Scientific, San Jose, CA, USA). The samples were loaded (5 μL/min) on a trapping column (Acclaim PepMap Nano Viper, C18, 3 μm, 75 μm × 150 mm, Thermo Scientific, Germany) with 0.1% FA. The peptides were separated using a separation column (nanoEase MZ PST CSH, 130 A, C18 1.7 μm, 75 μm × 250 mm, Waters, Germany), a flow rate of 300 nL/min and a gradient from 2% to 30% B (buffer A: 0.1% FA in HPLC–H_2_O; buffer B: 0.1% FA in ACN) in 60 min. The electrospray was generated from a steel emitter (Fisher Scientific, Germany) at a capillary voltage of 1850 V. MS/MS measurements were carried out in data dependent acquisition mode (DDA) using an HCD collision energy of 30% and top‐speed scan mode. Every 3 s MS scan was performed over an m/z range from 400 to 1300, with a resolution of 120,000 fwhm at m/z 200 (maximum injection time = 120 ms, AGC target = 2 × 10^5^). MS/MS spectra were recorded in the ion trap (rapid scan mode, maximum injection time = 60 ms, AGC target = 1 × 10^4^, quadrupole isolation width: 1.6 Da, intensity threshold: 1 × 10^4^). Precursors were excluded from DDA analysis for 60 s.

### 2.15 Protein identification and label free quantification

LC–MS/MS raw files were analyzed with ProteomeDiscoverer 2.4 (Thermo Scientific, San Jose, USA). For peptide and protein identification, the LC–MS/MS data were searched with SequesHT against a mouse database (SwissProt, 17,023 entries) and a contaminant database (116 entries). The following parameters were used for the data‐base search: mass tolerance MS1: 8 ppm, mass tolerance MS2: 0.5 Da, fixed modification: carbamidomethylation (cystein), variable modification: oxidation (methionine), and deamidation (glutamine, asparagine), variable modification at protein N‐terminus: acetylation, methionine loss, methionine loss + acetylation. FDR was calculated using Percolator. For feature detection, Minora Feature Detection was used with default settings. For label free quantification, the Precursor Ions Quantifier was used with the following parameters: Peptides to use: unique peptides, Precursor Abundance Based On: Area, Minimum Replicate Features: 50%, Normalization Mode: Total Peptide Amount, Protein Abundance Calculation: Summed Abundances.

### 2.16 Bioinformatics data processing and statistical analysis of the proteome data

The results of the LFQ analysis were exported from Proteome Discoverer as *.xlsx and further processed using R version 4.2.1 as describes by (Chen et al., 2020; Gardner & Freitas, 2021).

Proteins that did not contain quantitative values in at least three replicates in each condition, were removed from the data set. For proteins quantified in all replicates of one condition group, but not quantified in a single replicate of the other condition, missing values were replaced by a value of 2 to allow logarithmic transformation. Missing values for the other proteins were imputed using the *mice* function of the R package *mice* (van Buuren & Groothuis‐Oudshoorn, 2011) using the following parameters: imputation method: mean, function: *maxit*, maximum number of iteration: 500. The resulting imputed data were then extracted using the *complete* function. The data‐set was log2 transformed using the *log2* function (R base package).

Statistical analysis was performed using the R package *LIMMA*. Therefore, a design matrix with an intercept and a contrast matrix was made between the wild‐type group and the heterzygote knocked‐out group. The different abundance ratio levels were determined using the *eBayes* function to calculate moderated t‐statistics (Smyth, 2023). Benjamini–Hochberg correction (FDR: 0.01) was performed to correct for multiple testing and calculate adjusted *p* values. The following thresholds were used to define significantly up‐ and downregulated proteins: fold change of 1.5 (log2(1.5)), adjusted *p* value of <0.05.

Significant up‐ and downregulated proteins were subjected to Gene Ontology (GO) enrichment (Harris et al., 2004) using Web‐Gestalt (Liao et al., 2019). For downregulated proteins, the focus was set on statistically significant GO terms associated with regulation of synapse activity (28 proteins identified), altered synaptic transmission (5 proteins identified), and translation (3 proteins identified). For upregulated proteins, the focus was set on statistically significant GO terms associated with regulation of synapse activity (27 proteins identified), altered neuronal morphology (8 proteins identified), and neuronal degeneration (5 proteins identified). The above mentioned proteins were subjected to a network analysis using StringDB (Szklarczyk et al., 2021).

## 3 RESULTS

### 3.1 Decline in memory consolidation and episodic memory in aged *Tsc2* ^*+/−*^ animals

**FIGURE 1:**
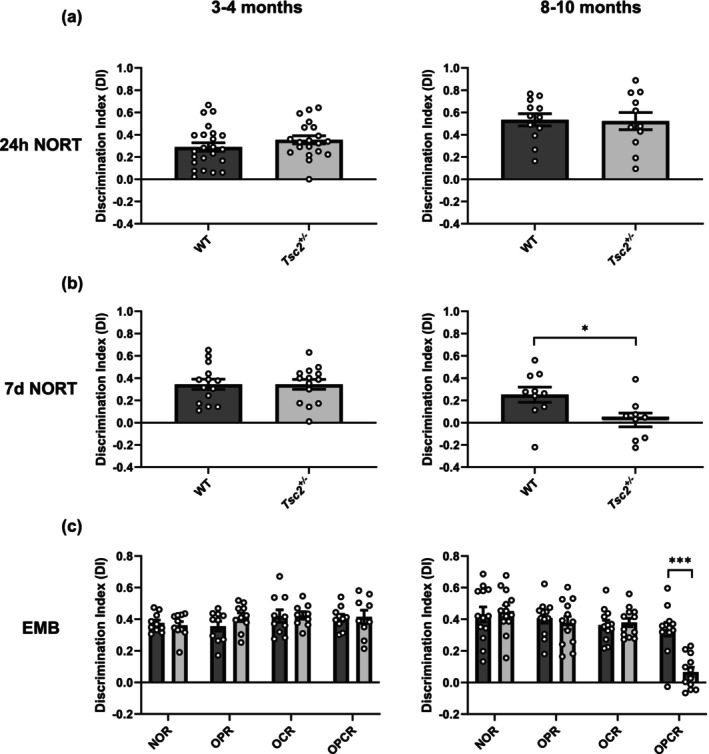
Cognitive decline in aged *Tsc2* ^+/−^ mice: (a) Mutants and wildtype controls are able to distinguish between familiar and novel objects in a 24 h NOR approach. Both 3 and 4 months old (left) and 8–10 months old (right) *Tsc2* ^*+/−*^ mice and their wildtype controls showed a significant preference for the novel object, as seen by the equally high discrimination index. (b) Increasing the interval between training and test phase to 7 days, 8–10 months old *Tsc2* ^*+/−*^ mice showed no preference between the novel and familiar object compared to wildtype controls (right; two‐tailed *t* test: *p* = 0.0254, *n*(WT) = 10, *n*(*Tsc2* ^*+/−*^) = 9), whereas both 3 and 4 months old *Tsc2* ^*+/−*^ *mice* and wildtype siblings still showed a significant preference (left; two‐tailed *t* test: *p* = 0.9941, *n*(WT) = 14, *n*(*Tsc2* ^*+/−*^) = 14) for the novel object. (c) The episodic memory battery (EMB) consists of four different NORT approaches (NOR, OPR, OCR, OPCR). The time the mice spent with the objects was measured during a test period of 2.5 min. *Tsc2* ^*+/−*^ mutants at 8–10 months of age (right; two‐tailed *t* test: *p* = 0.0000026, *n*(WT) = 10, *n*(*Tsc2* ^*+/−*^) = 9) were unable to perform the object‐location‐context recognition (OPCR) task, whereas they were able to recognize novel objects (NOR) and object‐location (OPR) and object‐context constellations (OCR). *Tsc2* ^*+/−*^ mutants at 3–4 months of age (left), as their wildtype siblings, were able to identify novel object constellation in all four tests. Discrimination index (DI) is calculated as: (time novel object − time familiar object)/(time new object + time familiar object). Values are means ± SEM, *p < 0.05, ***p < 0.001.

Mouse models with mutations in *Tsc1* or *Tsc2* exhibit a phenotypic profile similar to human patients, including cognitive impairments (Ehninger et al., 2008; Goorden et al., 2007). As cognitive impairments in TS patients develop over time, we performed a longitudinal analysis in *Tsc2* ^*+/−*^ males, using cohorts at 3–4 and 8–10 months of age. Contrary to what has been published previously, we did not find deficits in the Morris‐Water maze in both age groups tested (Figure S1). Additionally, a 24 h Novel Object Recognition approach revealed no aberrations in the *Tsc2* ^*+/−*^ mutants compared to wildtype controls in both age groups tested (Figure 1a). The object recognition test is a commonly used behavioral test to study various aspects of learning and memory in mice, taking advantage of the natural tendency of rodents to explore novelty. The test can be modified explicitly for numerous applications by either shortening the interval between training and testing phases to study short‐term memory or lengthening it to study long‐term memory and memory consolidation. In a test for novel object recognition with a prolonged phase of 7 days between the training and the testing phase, a significant reduction in performance was observed in 8–10 months old *Tsc2* ^*+/−*^ mutant mice compared to wildtype controls (Figure 1b), while no difference in exploration time was observed (Figure S2). No significant difference was seen in younger animals at 3–4 months of age. The specific impairment of the 7 days but not of the 24 h performance in 8–10 months old *Tsc2* ^*+/−*^ animals suggests a defect in memory consolidation with an onset in aging animals only. Episodic memory is another hippocampal‐based memory function. In a 4‐week test battery originally established to test episodic memory in rats, a significant reduction in performance in the episodic memory test (object‐place‐context recognition (OPCR) task) was found in 8–10 months old *Tsc2* ^*+/−*^ mice compared to wildtype controls (Figure 1c). By contrast, recognition of novel objects (NORT), object‐place (OPR), as well as object‐context constellations was not altered. No impairment in object‐place‐context recognition was observed in younger 3–4 months old *Tsc2* ^*+/−*^ mice. For each test, exploration times were comparable between groups. These data suggest an early degenerative decline in memory consolidation and episodic memory processing.

### 3.2 IEG expression in the brain

**FIGURE 2:**
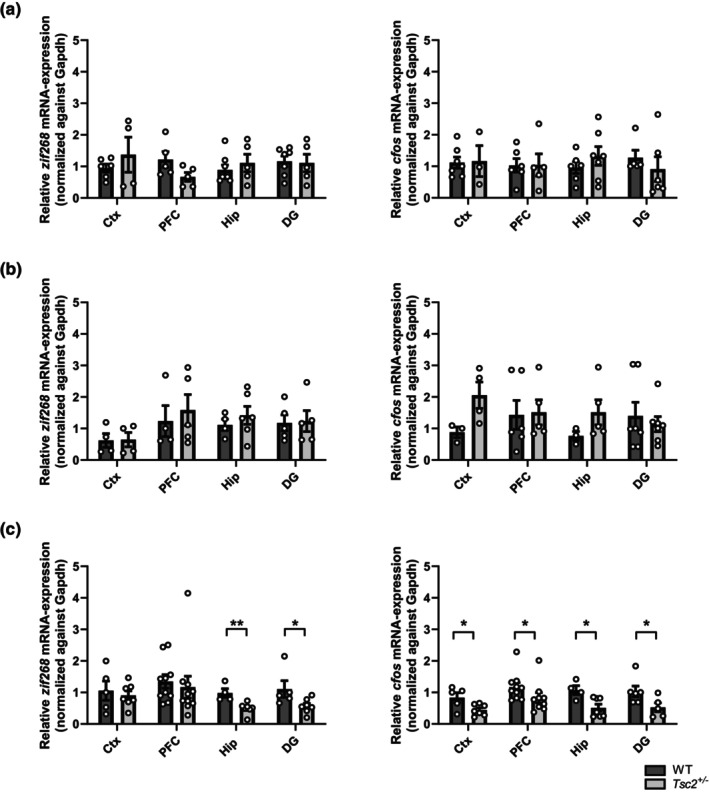
RT‐qPCR analysis of activity‐dependent IEGs *zif268* and *cfos* after training (a), 24 h NORT (b) and 7 d NORT (c). After training phase (a) and the 24 h NORT (b), RT‐qPCRs for *zif268* and *cfos* show no significant differences in brain regions of 8–10 months old *Tsc2* ^*+/−*^ mutants and wildtype controls. After 7 days NORT approach (c), relative *zif268* expression is significantly decreased in the Hippocampus of mutant mice compared to wildtype controls (left; two‐tailed *t* test: *p* = 0.005799, *n*(WT) = 4, *n*(*Tsc2* ^*+/−*^) = 7), whereas *cfos* expression level is significantly decreased in whole Cortex and Hippocampus compared to wildtype controls (right; *p*(Ctx) = 0.028450, *n*(WT) = 5, *n*(*Tsc2* ^*+/−*^) = 7); (*p*(Hip) = 0,014014, *n*(WT) = 4, *n*(*Tsc2* ^*+/−*^) = 7). (Ctx = cortex, pfc = prefrontal cortex, hip = hippocampus, DG = dentate gyrus; ct‐values were normalized against *gapdh* and are presented as means ± SEM, *p < 0.05, **p < 0.01).

To elucidate the mechanisms behind the early cognitive decline in *Tsc2* mutants, the expression of IEGs was studied in the context of deficient memory consolidation. The activity of IEGs in the brain is a proxy of neuronal activity. In particular, the expression of IEGs such as *cfos* or *zif268* is rapidly and selectively induced in subsets of neurons in specific brain regions associated with learning and memory formation (Minatohara et al., 2015; Tischmeyer & Grimm, 1999). In addition, it is known that dysregulation of mTOR impacts the expression of different IEGs (Murphy & Blenis, 2006). Expression patterns of the IEGs *cfos* and *zif268* were analyzed in the hippocampus, dentate gyrus, prefrontal cortex and whole cortex – the main structures of memory consolidation and episodic memory. Using quantitative real‐time PCR, it was shown that the expression of IEGs was significantly reduced in the *Tsc2* ^*+/−*^ mutants immediately after exposure to the new object in the test phase of the 7d NOR (Figure 2c), whereas no differences were observed after the training phase (Figure 2a) and after the 24 h NORT (Figure 2b). Specifically, the differences in *cfos* expression were seen in the hippocampus and whole cortex, whereas *zif268* expression was significantly decreased in the hippocampus of aged mutant *Tsc2* ^*+/−*^ mice.

#### 3.2.1 Hippocampal morphology in adult Tsc2^+/−^ animals

**FIGURE 3:**
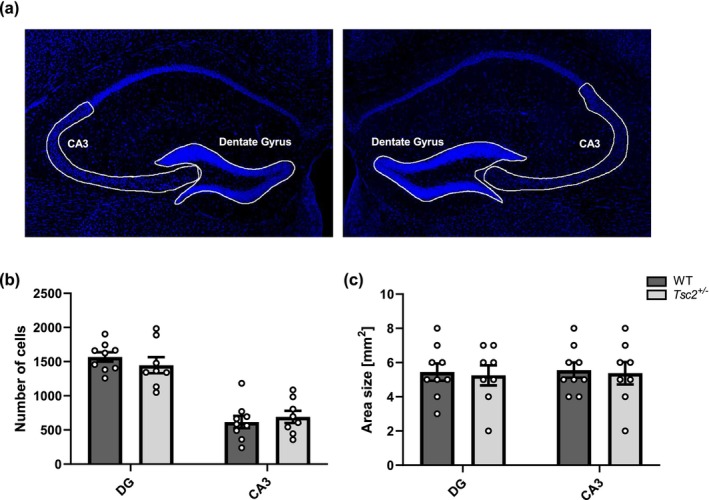
Hippocampal morphology of aged *Tsc2* ^*+/−*^ animals. (a) Confocal images of the dorsal hippocampus (exemplary wildtype mouse, both hemispheres) using nuclear staining (DAPI‐blue), in which the regions of the dentate gyrus (DG) and the cornu ammonis 3 (CA3) were defined as examples; nuclear staining was used to determine the cell count and area size in the defined regions. (b) and (c) showing the quantifications of cell count (b) and area size in mm^2^ (c) for the DG and CA3 in *Tsc2* ^*+/−*^ mutants and wildtype siblings at 8–10 months of age. There is no significant difference between *Tsc2+/−* mutants and wildtype siblings for any parameter examined or for either region (two‐tailed *t* test; *n*(WT) = 9, *n*(*Tsc2* ^*+/−*^) = 8). Quantification was based on the DAPI signal.

Searching for the mechanisms underlying the deficiency of hippocampal memory function and IEG expression, dorsal hippocampal neuroanatomy in *Tsc2* ^*+/−*^ mutants was examined for possible neurodegenerative processes potentially resulting in aberrant cell number or size of individual brain areas. As exemplified in Figure 3a, the areas of the dorsal dentate gyrus (DG) and cornu ammonis 3 (CA3) were manually defined based on the confocal images. Cells were counted semiautomatically using DAPI cell nuclear staining, and the area size of each brain area was calculated. Quantification of cell counts (Figure 3b) and area size (Figure 3c) for both the DG and CA3 regions shows no significant difference between *Tsc2* ^*+/−*^ mutants and wildtype controls for any of the parameters and regions examined. Thus, a macroscopic degenerative process in the hippocampus can be excluded as an underlying cause for the cognitive decline in aged *Tsc2* ^*+/−*^ mice.

### 3.3 Neurogenesis in adult *Tsc2* ^*+/−*^ animals

After the completion of postnatal development, remaining mammalian neural stem cells (NSCs) within the dorsal hippocampal dentate gyrus (DG) and the subventricular zone of the lateral ventricles are still capable of dividing into functional, adult born neurons throughout life (Kempermann et al., 2015). Although adult neurogenesis in humans is still under debate (Sorrells et al., 2018; Spalding et al., 2013), many studies indicate a strong relationship between functional adult neurogenesis and cognition (Toda et al., 2019). In order to quantify the impact of heterozygous deletion of *Tsc2* on adult neurogenesis, 10 months old *Tsc2* ^+/−^ mice and their corresponding wildtype littermates were injected with the Thymidine analogue BrdU. Since BrdU is incorporated into dividing cells, BrdU^+^ cells can be considered as adult born. We additionally used the neuronal marker NeuN as an explicit marker for mature neurons. Following this, adult born neurons were defined as BrdU/NeuN double positive cells (BrdU^+^/NeuN^+^). Immunohistochemical labelling and subsequent quantification of BrdU^+^/NeuN^+^ cells in coronal hippocampal brain sections revealed no alteration between *Tsc2* ^+/−^ and wildtype littermates (*p* = 0.57) (Figure S4i,p). Supportively, immunohistochemical analysis of the proliferation marker Ki67 revealed no quantitative difference between *Tsc2* ^+/−^ and wildtype littermates (*p* = 0.61) (Figure S4). Summarizing these findings, the heterozygous loss of *Tsc2* has no immediate impact on adult hippocampal neurogenesis.

### 3.4 Analysis of hippocampal projections in adult *Tsc2* ^*+/−*^ animals

Hippocampal memory function relies on neuronal circuits that connect the hippocampus with the entorhinal cortex on the one hand and the prefrontal cortex on the other. A malformation of hippocampal‐cortical projections could cause reduced expression of IEG in the hippocampus in response to memory challenges. In order to test this hypothesis, we used cholera toxin subunit B (CTB) as a retrograde tracer to map anatomical projections in the hippocampal formation and quantified the number of neurons projecting to the dentate gyrus in different brain areas (Aschauer et al., 2013). The tracer CTB is taken up by axon terminals in the injection sites, namely the dorsal and ventral dentate gyrus (Figure S5a–d), and transported back to the neuron's cell body, where neurons can be easily identified and counted. This allowed us to investigate potential differences in the functional organization between the hippocampal formation and connected brain areas in *Tsc2* ^*+/−*^ mice. We identified projection neurons in several brain areas, including well‐known ipsi‐ and contralateral connections from the CA3 and entorhinal cortex, as well as the supramammillary nucleus in the hypothalamus (Figure S5e–h) (Pan & McNaughton, 2004). Comparing absolute numbers of projection neurons to the dentate gyrus revealed no significant differences between wildtype and *Tsc2* ^*+/−*^ mice (Figure S5i–l). All connections between brain areas traced in wildtype mice were also observed in the heterozygous *Tsc2* ^*+/−*^ mice. This result indicates that hippocampal projections do not show major disturbances at the anatomical level in heterozygous knockout animals.

### 3.5 Synaptic and dendritic spine densities in the dorsal hippocampus of adult *Tsc2* animals

**FIGURE 4:**
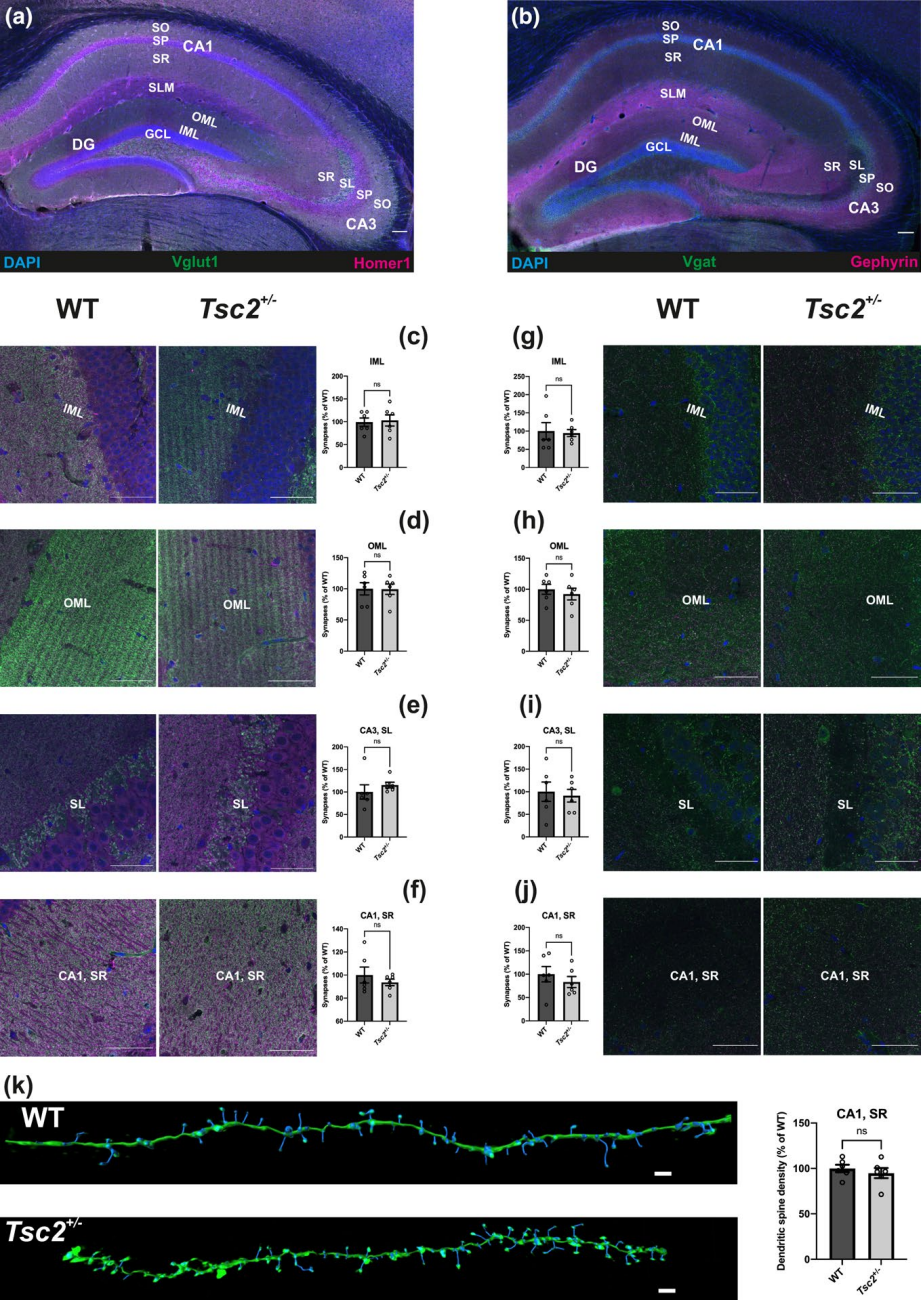
Analysis of synaptic and dendritic spine densities in the dorsal hippocampus of 9 months old *Tsc2* mice. Overview of the dorsal hippocampus stained with DAPI and excitatory pre‐ (Vglut1) and postsynaptic (Homer1) markers (a) or with DAPI and inhibitory pre‐ (Vgat) and postsynaptic (Gephyrin) markers (b). SO = stratum oriens, SP = stratum pyramidale, SR = stratum radiatum, SL = stratum lucidum, SLM = stratum lacunosum‐moleculare, GCL = granule cell layer, IML = inner molecular layer, OML = outer molecular layer. CA1 = Cornu Ammonis, area 1, CA3 = Cornu Ammonis area 3. Scale bar: 0.1 mm. Representative images of the inner molecular layer (c), outer molecular layer (d), stratum lucidum of CA3 (e) and stratum radiatum of CA1 (f) stained with the excitatory markers Vglut1 (presynaptic) and Homer1 (postsynaptic) in *Tsc2* wildtype (left, *n* = 6) and *Tsc2* ^*+/−*^ mice (middle, *n* = 6). Scale bars: 50 μm. Quantification of the excitatory synaptic number showed no change in the inner molecular layer (*p* = 0.8113) (c), outer molecular layer (*p* = 0.961) (d), stratum lucidum of CA3 (*p* = 0.3925) (e) and stratum radiatum of CA1 (f). Representative images of the inner molecular layer (g), outer molecular layer (h), stratum lucidum of CA3 (i) and stratum radiatum of CA1 (j) stained with the inhibitory markers Vgat (presynaptic) and Gephyrin (postsynaptic) in *Tsc2* wildtype (left, *n* = 6) and *Tsc2* ^*+/−*^ mice (middle, *n* = 6). Scale bars: 50 μm. Quantification of the inhibitory synaptic number showed no change in the inner molecular layer (*p* = 0.833) (g), outer molecular layer (*p* = 0.5478) (h), stratum lucidum of CA3 (*p* = 0.7353) (i) and stratum radiatum of CA1 (*p* = 0.4244) (j). (k) Exemplary reconstructions of apical dendrites from the stratum radiatum of CA1 in *Tsc2* wildtype (above, *n* = 6) and *Tsc2* ^*+/−*^ mice (below, *n* = 6). Scale bars: 2 μm. Analysis of the density of dendritic spine gave no significant change (*p* = 0.4721).

The lack of hippocampal morphological and projection correlates underlying the early memory deterioration in *Tsc2* ^*+/−*^ mice led us to hypothesize that more subtle synaptic or dendritic alterations could be responsible for the behavioral phenotype. In order to have an estimation of the number of excitatory and inhibitory synapses, immunohistochemical staining using pre‐ and postsynaptic excitatory and inhibitory markers, respectively, was performed as already shown in Rosales Jubal et al. (2021) (Figure 4a,b). Within the dorsal hippocampus, the subregions chosen are fundamental relay stations in the trisynaptic pathway. The entorhinal cortex projects both to the inner molecular layer (Deller et al., 1996) and outer molecular layer (Tamamaki & Nojyo, 1993) of the dentate gyrus. The dentate gyrus granule cells are further connected to the CA3 area of the hippocampus through synapses located in the stratum lucidum (Acsady et al., 1998). Finally, neurons located in the CA3 establish synapses with the neurons of the CA1 area, in the strata radiatum and oriens (Gulyas et al., 1993). The analysis of excitatory and inhibitory synapses in the regions mentioned above showed no significant difference between *Tsc2* wildtype and *Tsc2* ^*+/−*^ mice (Figure 4c–j). Also, the abundance of dendritic spines in the secondary dendrites of the CA1 stratum radiatum disclosed no significance difference (Figure 4k), suggesting that other mechanisms might be involved in the precocious memory decline of the *Tsc2* ^*+/−*^ mice.

### 3.6 Hippocampal proteomics

Since hippocampal morphology and projections did not show a significant change in 8–10 months old *Tsc2* ^*+/−*^ animals, we used hippocampal synaptosomes of *these* animals compared to wildtype littermates for a mass spectrometry analysis in order to identify changes at the molecular level that are causative for the observed cognitive restrictions in old *Tsc2* ^*+/−*^ animals.

**FIGURE 5:**
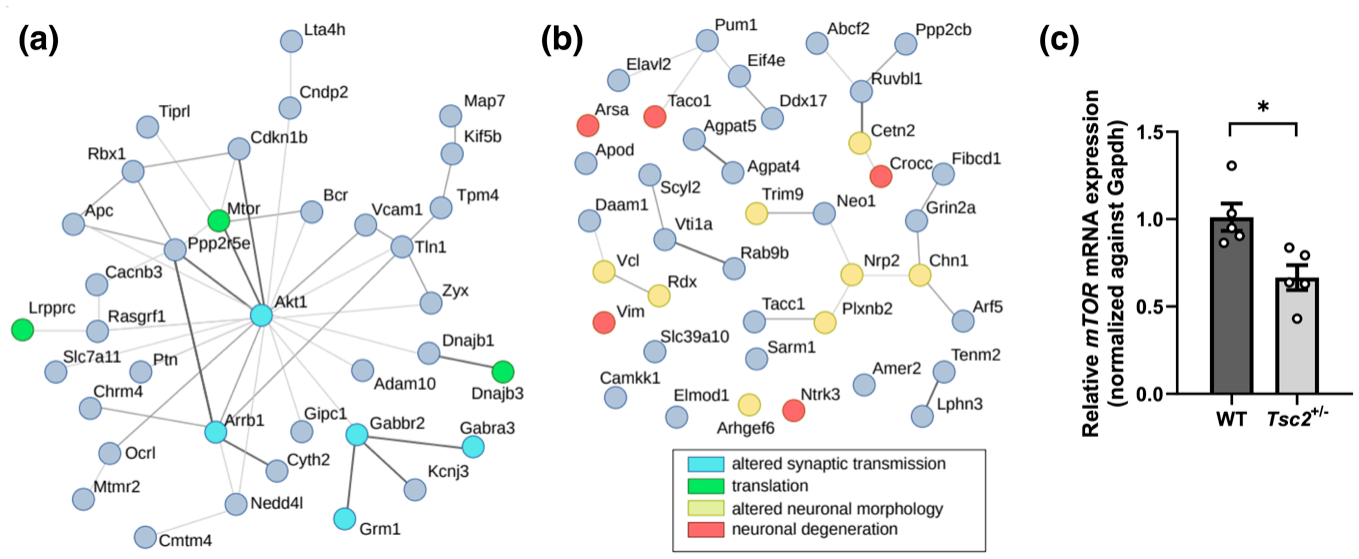
Network of significant hippocampal proteins displaying enriched GO‐Terms and showing downregulation of mTOR. Mass spectrometry analyzed hippocampal synaptosome data were filtered for significance; threshold used to define significantly up‐ and downregulated proteins were fold change of 1.5 (log2(1.5)) and adjusted *p* value of <0.05. Networks were created via StringDB and GO Term Enrichement Analysis was done via WebGestalt. The enriched GO‐Terms, found within the significant dataset, are displayed in the legend. (a) Network of downregulated hippocampal proteins. Blue nodes mark proteins, that show enriched GO‐Terms of altered synaptic transmission; green nodes mark proteins, that show enriched GO Terms regarding translation. Graph (b) shows network of upregulated hippocampal proteins. Yellow nodes mark proteins with enriched GO‐Terms regarding altered neuronal morphology and red nodes mark proteins with enriched GO Terms of neuronal degeneration. (c) RT‐qPCR‐analysis confirmed mTOR‐downregulation on mRNA level in hippocampal tissue of 10 months old *Tsc2* ^*+/−*^ animals compared to wildtype littermates (two tailed *t* test: *p* = 0.0117, *n*(WT) = 5, *n*(*Tsc2* ^*+/−*^) = 5). ct‐values were normalized against *gapdh* and are presented as means ± SEM, *p < 0.05.

GO term analysis of the results showed an upregulation of proteins associated with neuronal degeneration and morphology in hippocampal synaptosomes of 8–10 months old *Tsc2* ^*+/−*^ animals suggesting degenerative processes in the hippocampal synapses (Figure 5b). On the other hand, GO terms linked to synaptic transmission and protein translation were down‐regulated (Figure 5a). Protein‐network‐analysis confirmed this and revealed that it is particularly the Akt and mTOR kinases that are affected by the misregulation. Akt / mTOR play a fundamental role in the local synthesis of synaptic proteins. In line with this, network analysis showed that many of the proteins downregulated in the *Tsc2* ^*+/−*^ animals are linked through Akt and mTOR. Most important connections are between Akt and GABBR2, a Type B GABA receptor, Akt and β‐arrestin (Arrb1), which in concert with Ocrl (Inositol polyphosphate‐5‐phosphatase), Chrm4 (Colinergic receptor, Muscarinic, 4) and Nedd4l (ubiquitin protein ligase Nedd4 like) regulates receptor sensitivity and endocytosis (Buchanan et al., 2006; Chen et al., 2001; Noakes et al., 2011; Swan et al., 2010) and mTOR/Akt and Cacnb3, a voltage‐dependent calcium channel subunit.

Interestingly, while, as expected in heterozygous *Tsc2* knock‐out animals, the *Tsc2* mRNA was downregulated in hippocampal tissue of 10 months old animals, Tsc2 protein was not changed compared to wildtype littermates neither in hippocampal homogenates nor in synaptosomes of animals of the same age. This suggests post‐transcriptional compensatory processes in aging animals (Figure S6).

A down‐regulation of Akt/mTOR in aged *Tsc2* ^*+/−*^ animals is contrary to what has been observed in young *Tsc2* ^*+/−*^ animals, in which heterozygous loss of the mTOR inhibitor *Tsc2* results in mTOR hyperactivity (Ehninger et al., 2008). Our data suggests a reactive down‐regulation of Akt–mTOR in aging animals in response to continuous hyperactivity during development and adulthood. In order to confirm an effect on mTOR expression in aging *Tsc2* ^*+/−*^ animals we performed qRT‐PCR of the *mTOR* mRNA (Figure 5c) in hippocampal tissue of 10 months old animals. It was found significantly downregulated in the *Tsc2* ^*+/−*^ animals compared to wildtype littermates supporting a decrease in mTOR abundance despite *Tsc2* dysregulation in aging *Tsc2* ^*+/−*^ animals.

### 3.7 IGF2 injections rescue premature cognitive decline and IEG expression

**FIGURE 6:**
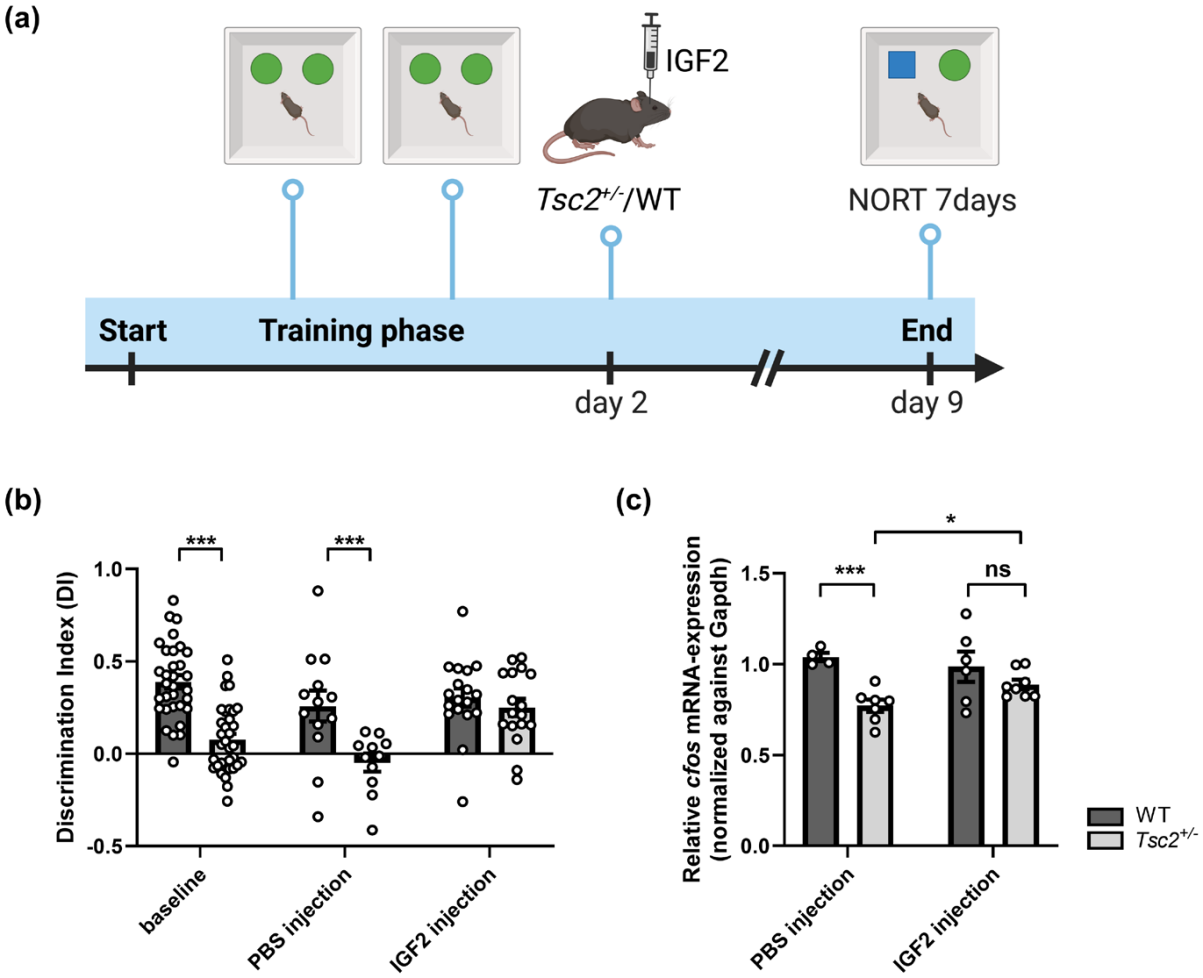
Hippocampal administration of recombinant IGF2 normalizes both memory consolidation and relative *cfos* expression in aged *Tsc2* ^*+/−*^ mice. (a) Schematic overview of 7d NORT including IGF2 administration on day 2 within 2 h after second training phase. (b), 7d NORT approach of aged *Tsc2* mutant mice 7 days after IGF2 injection showed a significant enhancement of memory consolidation measured as discrimination index compared to the PBS‐injected mutant mice (two tailed *t* test: *P*(baseline) = 5,89e‐9, *n*(WT) = 32, *n*(*Tsc2+/−*) = 34); *p*(PBS) = 0.0066, *n*(WT) = 13, *n*(*Tsc2* ^*+/−*^) = 11; *p*(IGF2) = 0.4433, *n*(WT) = 18, *n*(*Tsc2* ^*+/−*^) = 17. (c) Relative mRNA expression levels of *cfos* in the hippocampus of aged *Tsc2* mutants after IGF2 administration compared to the control group. No significant difference in *Tsc2* ^*+/−*^ mice compared to wildtype controls after IGF2 treatment (two‐tailed *t* test: *p*(PBS) = 0.0004, *n*(WT) = 4, *n*(*Tsc2* ^*+/−*^) = 7); *p*(IGF2) = 0.2329, *n*(WT) = 6, *n*(*Tsc2* ^*+/−*^) = 8. Values were normalized against Gapdh and are presented as mean ± SEM, *p < 0.05, ***p < 0.001. Quantification was performed using Excel.

IGF2 is a ligand of the insulin receptor cascade and is tightly connected to the mTOR signaling cascade as a stimulator of insulin/mTOR signaling (summarized in Bergman et al., 2013) and at the same time, in a feedback loop, in which both, its transcription and its translation, has been found to be a target of mTOR (Dai et al., 2011; Ge & Chen, 2012). Furthermore, it has been shown to increase spine maturation and enhance memory consolidation in the mouse hippocampus by activating the insulin/mTOR signaling cascade (Chen et al., 2011; Schmeisser et al., 2012; Steinmetz et al., 2018; Stern et al., 2014). qRT‐PCR of the *Igf2* mRNA on hippocampal RNA of aging *Tsc2* ^*+/−*^ animals revealed a significant downregulation of *Igf2* compared to wildtype littermates suggesting a direct effect of mTOR dysregulation on *Igf2* expression (Figure S7). In order to further validate the influence of Akt/mTOR/IGF2 downregulation on the cognitive decline phenotype, we investigated if IGF2 injection can rescue memory consolidation in aged *Tsc2* ^*+/−*^ mice. Recombinant IGF2 (or PBS as a vehicle control) was injected bilaterally into the dorsal hippocampus of *Tsc2* ^*+/−*^ animals and wildtype littermates immediately after the second training of the 7d NORT (Figure 6a). Seven days after injection, animals were tested with a new object in the test phase of the 7d NORT and exploration times were measured. The discrimination indices of the *Tsc2* ^*+/−*^ animals showed a significant increase after IGF2 injection compared to the vehicle control, reaching the level of wildtype control animals (Figure 6b).

Recently, Yu et al. had demonstrated an effect of IGF2 on transcription of IEGs and training‐dependent increase in de novo protein synthesis via activation of the IGF2 receptor in addition to improving memory performance (Yu et al., 2020). Since we have already demonstrated altered training‐dependent IEG expression in the hippocampus of old *Tsc2* ^*+/−*^ animals, we aimed at analyzing the effect of IGF2 on the expression of the IEG *cfos* in the hippocampus. *Tsc2* ^*+/−*^ animals and wildtype littermates were exposed to the 7d NORT together with bilateral IGF2 injection or vehicle injection into the dorsal hippocampus, respectively. Within 60 min after testing on day 9, hippocampi were isolated and RNA was extracted. By using qRT‐PCR, the expression of the IEG *cfos* in the hippocampus, in both IGF2 and vehicle‐injected animals, was examined. As depicted in Figure 6c, a significant increase in *cfos* mRNA expression was detected after IGF2 treatment in *Tsc2* mutants compared to vehicle‐treated *Tsc2* mutants. In conclusion, the growth factor IGF2 has a positive effect on memory performance and on the training‐dependent expression of the IEG *cfos* in the hippocampus in aged *Tsc2* ^*+/−*^ mice.

## 4 DISCUSSION

We here have studied a non‐inducible knock‐out mouse model for TS carrying a heterozygous deletion in the *Tsc2* gene. As also observed in patients we found a sequential assembly of symptoms of the TAND spectrum. We have put a particular focus on the trajectory of cognitive (dis)abilities in the *Tsc2* ^*+/−*^ animals and found that the infantile, adolescent and young adult animals did not show any impairment of cognitive abilities, whereas aging animals exhibited a subtle deterioration of hippocampus‐based memory function. Molecular workup demonstrated a substantial decline of IEG expression after memory challenge in the hippocampus and a decrease of mTOR signaling cascade expression in hippocampal synaptosomes of aged *Tsc2* ^*+/−*^ mice. Stereotactic hippocampal injection of IGF2, a ligand that induces mTOR signaling through the insulin growth factor receptors could fully rescue the behavioral phenotype as well as the aberrant expression of IEG expression after challenge, supporting occurrence of premature erosion of the mTOR signaling pathway in *Tsc2* ^*+/−*^ animals caused by continuous over‐activity over lifetime.

Autism‐like features could be, as shown previously (Ehninger et al., 2008), observed in 2–4 months old, heterozygous *Tsc2* ^*+/−*^ animals. However, we did not detect cognitive impairment in infantile, adolescent or young adult *Tsc2* ^*+/−*^ animals. This is discrepant to the observations of other groups (Ehninger et al., 2008) and might point towards a critical influence of environmental factors caused by divergent husbandry conditions in the animal facilities on the development of the cognitive phenotype in young *Tsc2* ^*+/−*^ animals. The clinical variability seen in TS patients could be the human counterpart of this observation in animal behavior. Further work to investigate the influence of, for example, light, temperature, and food conditions as well as numerous other stress factors in the different facilities will be important to prove this hypothesis. Deeper insight into environmental factors influencing clinical phenotype expression could become an important factor for preventive treatment strategies in young children with the molecular diagnosis of TS.

The premature cognitive decline that we have observed, however, has not been described in animals or patients before, probably because longitudinal analysis of cognitive function in *Tsc2* ^*+/−*^ animals has not been performed until old age so far. Our observations point towards a substantial influence of developmental signatures on the aging nervous system.

A close relation between neurodevelopmental impairment and premature cognitive decline was first recognized in Down syndrome. Patients with Down syndrome not only show premature aging of many organs but also develop neurodegeneration and Alzheimer syndrome‐like features as early as in the 4th decade of life (summarized in Zigman, 2013). This observation was extended to patients with other types of intellectual disability (ID) by the finding of an inverse association between cognitive reserve in middle age and neurodegeneration (Ferreira et al., 2017) and the observation of early cognitive decline in patients with ID (Batty et al., 2007; Kilgour et al., 2010). In line with that, in Cornelia de Lange syndrome patients, a syndromic form of ID combined with dysmorphic and autistic features, premature brain aging has been found (Kline et al., 2007). In patients with ADHD (attention deficit hyperactivity disorder) brain regions undergoing pathologically late maturation were shown to degenerate abnormally early (Kakuszi et al., 2020) and patients with schizophrenia, which has its roots in an impaired neurodevelopment also show premature decline of brain function (Nenadic et al., 2017). Furthermore, also autism spectrum disorder (ASD) seems to be associated with early cognitive decline. Animal experiments show that knock‐out of Dock4, a gene that is closely associated to autism, leads to loss of hippocampal‐based spatial memory significantly earlier than in the control group (Guo et al., 2022).

Mechanistically, immunological aberrations were found in patients with ID and ASD (Di Marco et al., 2016; Gensous et al., 2020) and inflammatory processes have been suggested to link ID with premature cognitive decline (Carmeli et al., 2012). However, beyond that, molecular mechanisms underlying premature cognitive decline following neurodevelopmental impairment are largely unknown. Here, we found that a substantial decrease of IEG expression during memory challenge and molecularly, an increase of markers of neurodegeneration and a decrease of expression of components of the mTOR/Akt1 signaling cascade are associated with the cognitive decline in aging *Tsc2* ^*+/−*^ animals. Since loss of *Tsc2* function leads to hyperactivity of the mTOR pathway in young animals (Ehninger et al., 2008), our data suggest synaptic abrasion of the mTOR / Akt1 pathway together with an upregulation of markerproteins for neurodegeneration caused by an increased consumption of mTOR activity across lifetime.

The mTOR kinase is a key regulator of local protein synthesis at the synapse and thereby plays an important role in training regulated protein metabolism in the postsynaptic compartment, which is essential for memory consolidation (summarized in Richter & Klann, 2009). mTOR inhibition through rapamycin or metformin in young years has been demonstrated to increase life‐span and also to enhance cognitive abilities later in life (summarized in Aiello et al., 2022; Madhu et al., 2022; Santos et al., 2011; Triggle et al., 2022; Weichhart, 2018). However, in a recent paper, the positive effects of metformin on life‐span of *C.elegans* were found to only kick in when metformin is given early in life (Espada et al., 2020). When given late it has an apparently toxic effect, significantly affecting longevity. Our data shows that a continuously increased mTOR activity in a *Tsc2* ^*+/−*^ animal model with time leads to a substantial downregulation of molecules of the mTOR pathway and to premature cognitive decline. When, at this stage, mTOR activity is further suppressed, a negative effect is plausible. We think that what we see in *Tsc2* haploinsufficiency is the extreme end of a crescendo of mTOR activity during lifetime, that is indirectly proportional to cognitive health in individuals and is associated with a reduced ability of the mTOR signaling cascade to react to activity stimulation in the synapse during aging. In line with this, reduced mTOR activity restricted to the synapse has been found to be associated with impairment of activity dependent protein synthesis in Alzheimer's disease mice (Ahmad et al., 2017). It has been observed that transient treatment with rapamycin during early stages of development in mouse and drosophila is able to reset the system and tremendously affect life‐span (Aiello et al., 2022). Our data suggests that early inhibition of mTOR might similarly be able to prevent overburdening of the synaptic mTOR signaling cascade and thereby abrasion of local protein synthesis and cognitive decline could be delayed in *Tsc2* ^*+/−*^ animals.

As we show here, an acute treatment using IGF2 supports mTOR stimulation in the synapse, thereby compensating for the mTOR erosion and rescuing the premature aging phenotype of hippocampal function and activity. IGF2 has been shown previously to enhance memory function and rescue autistic‐like behavior in mice (Chen et al., 2011; Steinmetz et al., 2018). In the hippocampus, IGF2 binds primarily to the IGF2 receptor and thereby stimulates mTOR/Akt1 signaling (Steinmetz et al., 2018). Our data suggests that, in a scenario of continuous erosion of synaptic mTOR activity, acute injection with IGF2 is the treatment of choice to postpone cognitive decline. Future research is necessary to further investigate the hypothesis that—not only in *Tsc2* ^*+/−*^ animals, but also during normal human aging—individuals with continuously high mTOR activity, will experience an erosion of the mTOR cascade leading to impairment of mTOR dependent protein synthesis at the synapse, thus leading to early cognitive impairment. This would make treatment with IGF2 a promising option to improving cognitive health in the aging population, which is supported by previous observations showing that AAV‐mediated overexpression of IGF2 in old mice rescued cognitive impairment (Pascual‐Lucas et al., 2014).

## AUTHOR CONTRIBUTIONS

S.S. and M.S. conceived and designed the project; J.K. and A.A. performed and analyzed behavioral‐ and qPCR‐experiments; J.K. and D.A. performed neuroanatomical tracing experiments, data were analyzed and interpreted by D.A. under supervision of S.R. and S.S. F.B., and J.K. performed IGF2 administration and J.K. analyzed the outcome data. M.D.K. and K.V. performed and analyzed the massspectrometry analysis together with R.S. C.C., and S.G. processed and interpreted the proteomic dataset. L.N. performed and analyzed synaptic and dendritic spine densities and J.M. performed neurogenesis experiments under supervision of M.S. K.R. provided initial training for the isolation of synaptosomes which were performed by J.K. V.E. analyzed *Tsc2* mRNA expression levels. S.S. M.S., and J.K. wrote the manuscript, with all authors contributing corrections and comments. Correspondence and requests for materials should be addressed to S.S.

## FUNDING INFORMATION

The work was funded by the Deutsche Forschungsgemeinschaft through the CRC 1080 to S.S., M.S. and S.R., the SPP 2041 to S.R., the DIP “Neurobiology of Forgetting” to S.R. and the Deutsche Forschungsgemeinschaft/Agence nationale de la recherche #431393205 to S.R.

## CONFLICT OF INTEREST STATEMENT

The authors declare no competing interests.

## Supporting information


Data S1.

## Data Availability

All data needed to evaluate the conclusions in the paper are present in the paper and/or the Supplementary Materials.
