# Supplementary material for: Premature cognitive decline in a mouse model of tuberous sclerosis
Source: Aging Cell. 2024 Aug 27;23(12):e14318. doi: 10.1111/acel.14318 (PMC11634721; doi:10.1111/acel.14318)
Supplement: Supplementary file 1 — Data S1. [file ACEL-23-e14318-s001.docx]

**Supplementary Material for article:**

**Premature cognitive decline in a mouse model of tuberous sclerosis**

J. Krummeich^1#*^, L. Nardi^2^, C. Caliendo^1^, D. Aschauer^3^, V. Engelhardt^1^, A. Arlt^1##^, J. Maier^2^, F. Bicker^2^, M. D. Kwiatkowski^4^, K. Rolski^4^, K. Vincze^4^, R. Schneider^4^, S. Rumpel^3^, S. Gerber^1^, M. J. Schmeisser^2^, S. Schweiger^1,5,6*^

1. Institute of Human Genetics, University Medical Center of the Johannes Gutenberg University Mainz, Mainz, Germany
2. Institute of Anatomy, Johannes Gutenberg University Mainz, Mainz, Germany
3. Institute of Physiology, Johannes Gutenberg University Mainz, Mainz, Germany
4. Department of Biochemistry, University of Innsbruck, Innsbruck, Austria
5. Leibniz Institute of Resilience Research, Mainz, Germany
6. Institute of Molecular Biology, Mainz, Germany

^#^present address: Institute of Pathology, University Medical Center, Mainz, Germany

^##^present address: Institute for Genomic Statistics and Bioinformatics, University of Bonn, Germany

*Correspondence: [jenniferkrummeich@uni-mainz.de](mailto:jenniferkrummeich@uni-mainz.de), [schweigs@uni-mainz.de](mailto:schweigs@uni-mainz.de)

**This PDF file includes:**

Figs. S1 to S7

**Supplementary Figure 1: Morris Water Maze**

Motivation to reach the target platform and memory acquisition were unaffected in *Tsc2*^+/-^ mutants of both age groups compared to wildtype controls. Mice were trained in a water-filled tank to locate a quadrant that contained a hidden platform (PQ), learning visual cues to navigate. The distance traveled to reach the visible platform (a) and the latency (b) was not different between *Tsc2*^+/-^ mice and wildtype controls, neither at 3-4 months of age (left) nor at 8-10 months of age (right). All groups were able to learn and remember to find the platform during the 8 days training phase. Ten days after Morris water maze training, time spent in the target quadrant (PQ) vs. other quadrants was measured in a test trial (c). *Tsc2*^+/-^ mutants of both age groups spent significantly more time in the target quadrant with the hidden platform compared to the other quadrants (PL, PR and PG), similar to wildtype controls.

**Supplementary Figure 2: Exploration time of 24 hour-/7days NORT**

Total exploration time of *Tsc2*^+/-^ mice and wildtype controls during 24 hour NORT (a) and 7days NORT (b) showed no significant difference between the genotypes.

**Supplementary Figure 3: Motor abilities using distance traveled-parameter in the Open Field Test**

The traveled distance of *Tsc2*^+/-^ mice and wildtype controls during a10-minutes Open Field Test shows no significant difference between the genotypes at neither of the two times, excluding motor impairments.

**Supplementary Figure 4: No quantitative change in adult hippocampal neurogenesis due to *Tsc2* haploinsufficiency**

a-d, Confocal example images of coronal hippocampal sections from WT-derived tissue. Arrowheads indicate NeuN+/BrdU+ (adult born neurons) cells. Scale bar: 50 µm. e-h, Coronal slices of *Tsc2*^+/-^ mice hippocampal tissue. Arrowheads indicate NeuN+/BrdU+ cells. Scale bar: 50 µm. (i) Ratio of BrdU+/NeuN+ cells in 10 months old mice (WT: 1.71 ± 0.42 cells/mm vs. *Tsc2*^+/-^: 1.36 ± 0.37 cells/mm, n=4, p=0.57). j-l, Coronal slices of WT mice hippocampal tissue. Arrowheads indicate Ki67+ cells. Scale bar: 50 µm. m-o, Coronal slices of *Tsc2*^+/-^ mice hippocampal tissue. Arrowheads indicate Ki67+ cells. Scale bar: 50 µm. (p) Ki67+ cells in the hippocampal DG of 10 months old mice (WT: 3.26 ± 0,95 cells/mm vs. *Tsc2*^+/-^ mice: 2.61 ± 0.78 cells/mm, n=4, p=0.61).

**Supplementary Figure 5: Anatomical mapping of hippocampal projections**

A, Confocal image of injection site of CTB-488 into dorsal Dentate Gyrus (DG) next to illustration from brain atlas (grey = DAPI, green = CTB, scale bar = 50 µm). b, Spread of CTB-injection in dorsal DG in each injected mouse, quantified as integrated fluorescence of CTB-coupled fluorophore. c and d, same as (a) and (b), for injection of CTB-555 into ventral DG (grey = DAPI, red = CTB, scale bar = 50 µm). e and f, Confocal image of contralateral dorsal DG from a wildtype and a *Tsc2^+/-^* mouse injected with CTB-488 into the dorsal DG and CTB-555 into the ventral DG. Green cells project to ipsilateral dorsal DG, while red cells project to ventral DG. Signal surrounding the granular cell layer comes from projection fibers. g and h, same as (e) and (f) for ventral DG. i Mean number of projection neurons in dorsal DG from respective area and genotype. There was no significant difference between the groups. j, Same as i for ventral DG. k, network diagram illustrating quantification of projection neurons to dorsal and ventral DG in all analyzed brain areas from experiments with wildtype mice (dDG: dorsal Dentate Gyrus, dCA3: dorsal CA3, vDG: ventral Dentate Gyrus, ECx: Entorhinal Cortex, SuM: Supramammillary Nucleus). Thickness of the arrows is proportional to the number of projection cells on a log scale (see the scale in the black box in the middle). i, Same as k, from experiments with *Tsc2*^+/-^ mice. After Bonferroni correction for multiple comparisons, there was no significant difference in any area.


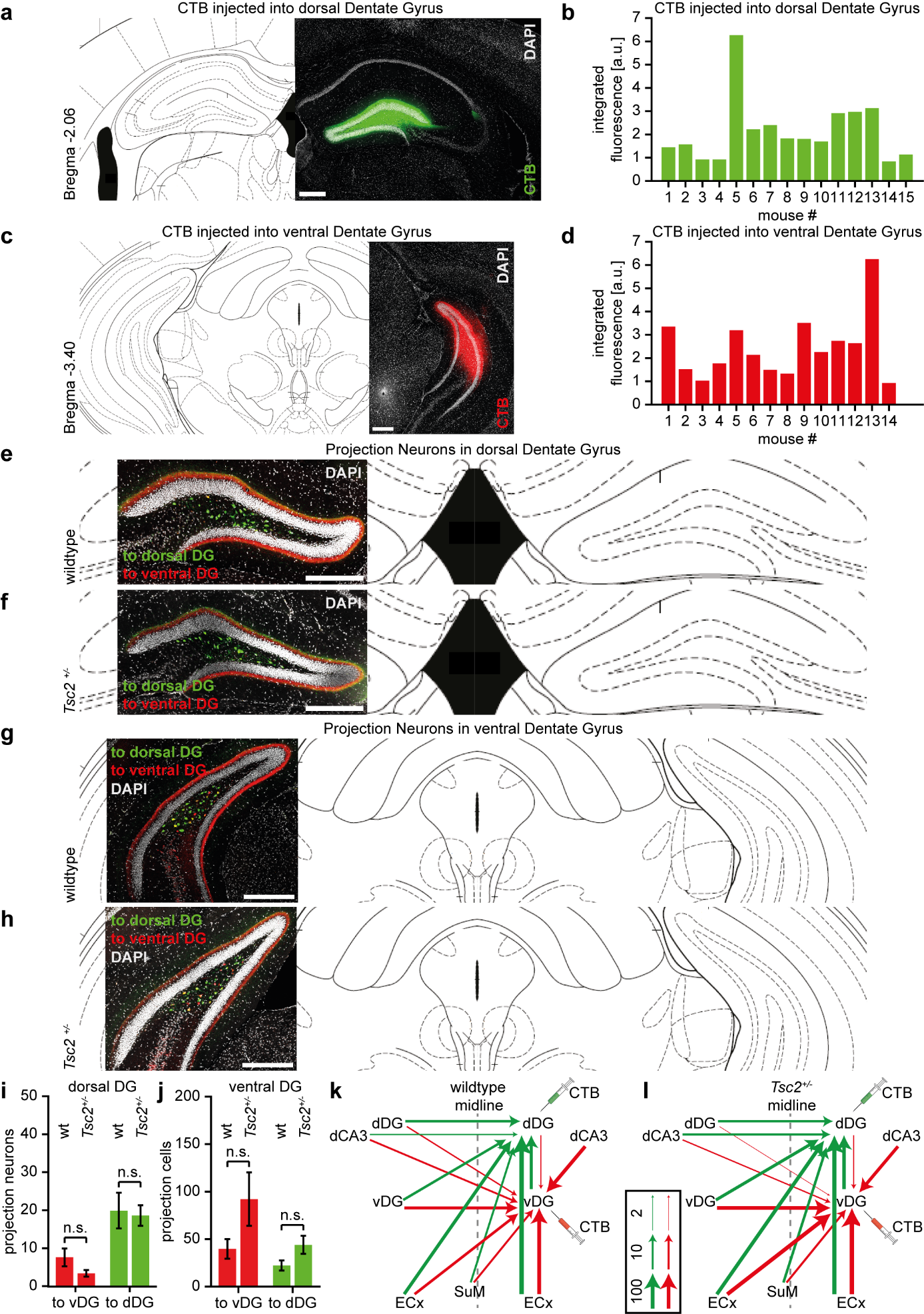


**Supplementary Figure 6: Downregulation of hippocampal Tsc2 mRNA levels but not protein expression in aged *Tsc2*^+/-^ mutant animals**

RT-qPCR results show significantly reduced *Tsc2* mRNA levels in the hippocampus of 10 months old *Tsc2*^+/-^ mutants compared to wildtype controls (two-tailed t-test: p=0.0002, n(WT)=5, n(*Tsc2*^+/-^)=5), whereas Tsc2 protein expression analyzed by Western blot is unaltered in hippocampal homogenates and synaptosomal fractions between 12 months old *Tsc2*^+/-^ mutant and wildtype controls (two-tailed t-test: p=0.63, p=0.93, n(WT)=6, n(*Tsc2*^+/-^)=6). Relative expression levels were normalized to *Gapdh* and are presented as means ± SEM, *p<0.05, **p<0.01, ***p<0.001.


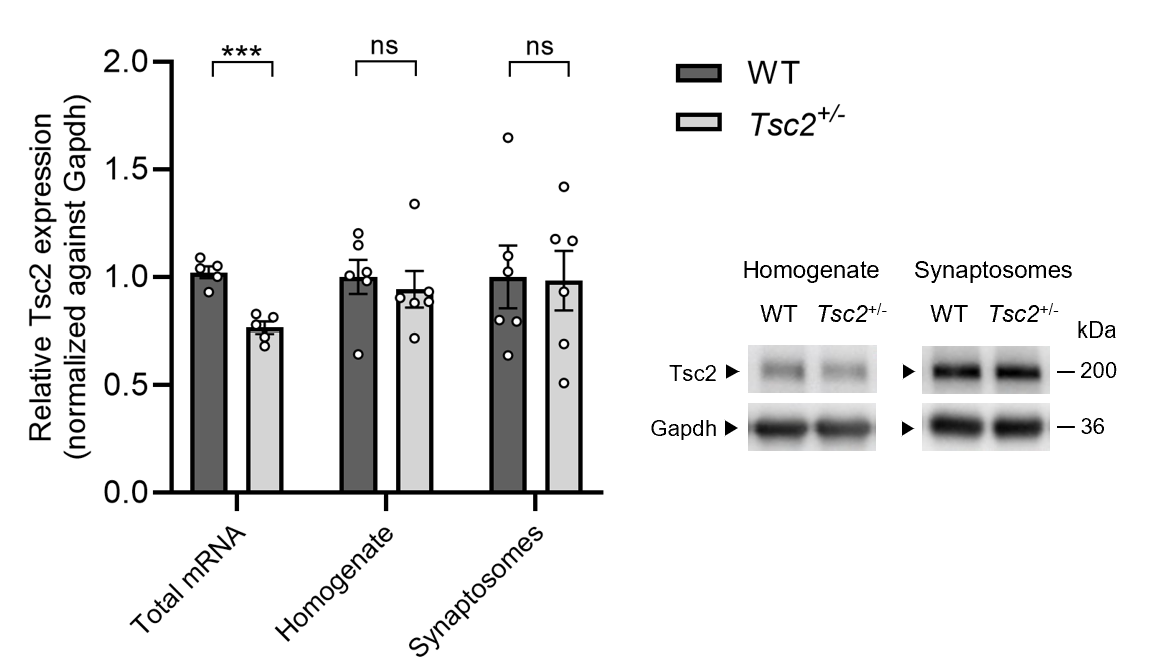


**Supplementary Figure 7: Downregulation of hippocampal *Igf2* mRNA levels in aged *Tsc2*^+/-^ mutant animals**

RT-qPCR results show significantly reduced *Igf2* mRNA levels in the hippocampus of 10 months old *Tsc2*^+/-^ mutants compared to wildtype controls (two-tailed t-test: p=0.027, n(WT)=5, n(*Tsc2*^+/-^)=5)
